# Noncoding RNA Roles in Pharmacogenomic Responses to Aspirin: New Molecular Mechanisms for an Old Drug

**DOI:** 10.1155/2021/6830560

**Published:** 2021-12-09

**Authors:** Mohammad Amin Khazeei Tabari, Mohammad Amir Mishan, Mona Moradi, Mohanna Khandan, Hooman Khoshhal, Abdolkarim Mahrooz, Abouzar Bagheri

**Affiliations:** ^1^Student Research Committee, Mazandaran University of Medical Sciences, Sari, Iran; ^2^USERN Office, Mazandaran University of Medical Sciences, Sari, Iran; ^3^Ocular Tissue Engineering Research Center, Research Institute for Ophthalmology and Vision Science, Shahid Beheshti University of Medical Sciences, Tehran, Iran; ^4^Department of Clinical Biochemistry and Medical Genetics, Faculty of Medicine, Molecular and Cell Biology Research Center, Mazandaran University of Medical Sciences, Sari, Iran; ^5^Department of Clinical Biochemistry and Medical Genetics, Gastrointestinal Cancer Research Center, Faculty of Medicine, Mazandaran University of Medical Sciences, Sari, Iran

## Abstract

Aspirin, as one of the most frequently prescribed drugs, can have therapeutic effects on different conditions such as cardiovascular and metabolic disorders and malignancies. The effects of this common cardiovascular drug are exerted through different molecular and cellular pathways. Altered noncoding RNA (ncRNA) expression profiles during aspirin treatments indicate a close relationship between these regulatory molecules and aspirin effects through regulating gene expressions. A better understanding of the molecular networks contributing to aspirin efficacy would help optimize efficient therapies for this very popular drug. This review is aimed at discussing and highlighting the identified interactions between aspirin and ncRNAs and their targeting pathways and better understanding pharmacogenetic responses to aspirin.

## 1. Introduction

Acetylsalicylic acid (ASA), generally known as aspirin, is mostly prescribed for treating patients with cardiovascular diseases [1]; besides, it can also have therapeutic effects on the different types of cancers and metabolic diseases [2, 3] by modulating different molecules and cellular signaling pathways [4].

Noncoding RNAs (ncRNAs) are regulatory RNAs that could modulate different steps in the transcription and translation processes [5–7]. ncRNAs are powerful, flexible, and pervasive cellular regulators. They are among the most critical molecules that aspirin can affect and subsequently cause many changes in the cellular signaling pathways [8, 9]. ncRNAs have different classifications, but so far, the effects of aspirin have been reported just on the microRNAs (miRNAs, miRs) and long noncoding RNAs (lncRNAs) [9, 10].

The discovery of ncRNAs has changed our understanding of the biology of diseases. A better knowledge of the interactions between ncRNAs and drugs can help clarify the molecular mechanisms by which drugs exert their effects. Some previous review studies clarified the effects of aspirin on miRNAs in cardiovascular diseases [11] and cancer [12]; however, none investigated the effects of aspirin on ncRNAs in different diseases. This review intends to discuss aspirin effects on ncRNAs to identify their impacts in detail and elucidate potential therapeutic approaches.

### 1.1. Aspirin: Sources, Bioavailability, and Mechanism of Action

Aspirin is a nonsteroidal anti-inflammatory drug (NSAID), which is mostly used against platelet aggregation and pain, which has inhibitory activities in various disorders such as cancers and cardiovascular and central nervous system (CNS) disorders [13, 14]. Aspirin's molecular formula is C_9_H_8_O_4_, and its IUPAC name is “2-acetyloxybenzoic acid” [15].

It is believed that aspirin naturally originated from willow bark. Willow species have small amounts of salicin, which would be turned into salicylate fractions. Salicylic consistency is higher in aspirin pills than willow bark, and willow bark cannot be a suitable source for analgesia alone [14, 16]. Prescription and dose of aspirin can vary among different diseases, from 50 mg to 6000 mg daily. Most of its side effects, such as gastrointestinal bleeding, are dose-dependent [17]. An investigation indicated that after administering a 100 mg dose of aspirin, the average *C*_max_ was 0.94 mg/L and 2 mg/L in patients with myocardial infarction and healthy people, respectively [18].

It is believed that aspirin's analgesic and antiplatelet activity is due to the ability of this drug to lower prostaglandins and thromboxane A2 [19]. Aspirin can inhibit prostaglandins and thromboxane due to its ability to suppress cyclooxygenase (COX). COX is needed to produce prostaglandins and thromboxane. Aspirin is an acetylating compound that can bind to the serine residue of COX. Thus, aspirin inhibits the enzyme irreversibly, which is different from the other NSAIDS that are reversible inhibitors. COX-1 suppression leads to thromboxane repression and vasoconstriction. COX-2 suppression also causes prostaglandin inhibition and, consequently, inflammation prevention [16, 20]. Aspirin can also be involved in uncoupling oxidative phosphorylation in mitochondria resulting in a higher respiration rate and diminished respiratory control ratio and signaling modulation through preventing NF-*κ*B in neoplastic cells [21, 22].

### 1.2. ncRNAs

Most of the mammalian transcriptomes are noncoding [23]. ncRNAs are divided into two categories, regulators and housekeepers [24]. So far, different classes of regulatory ncRNAs have been found in mammalians that have significant roles in most cellular signaling pathways [25, 26] (Table 1).

**Figure 1:**
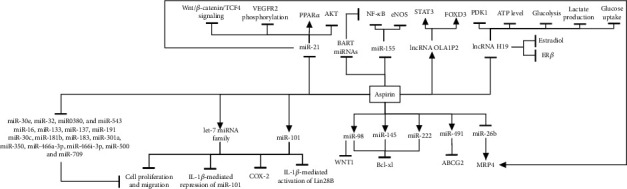
Identified effects of aspirin on ncRNAs. Aspirin alters the expression of miRNAs and lncRNAs and subsequently their targets.

**Figure 2:**
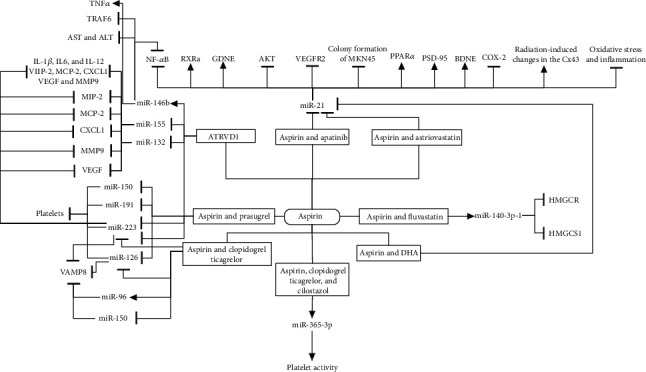
Effects of aspirin combined with other drugs on ncRNAs. Aspirin combined with other drugs alters the expression of miRNAs and lncRNAs and consequently their targets.

Regulatory ncRNAs are involved in gene expression regulation under physiological and pathophysiological conditions [27, 28]. So far, the effects of aspirin alone or with others on the two classes of ncRNAs, including lncRNAs and miRNAs, have been reported (Figures 1 and 2). lncRNAs have regulatory effects on the transcriptional and posttranscriptional stages [29]. They play essential roles in biological activities and participate in many disorders, especially in cancer and chronic diseases [30–34]. Apart from the gene expression's effect, lncRNAs can also stick to proteins and modulate their necessary functions for signaling pathways [35]. Among ncRNAs, miRNAs are the main agents for gene silencing and posttranscriptional regulation. These molecules affect gene expression by attaching to specific areas in the UTRs or coding regions of the targets and impressing RNA expression or function [36–38].

## 2. Effects of Aspirin on ncRNAs in Different Conditions

The effects of aspirin on ncRNAs in various conditions are demonstrated as follows and briefly in Table 2.

### 2.1. Osteosarcoma

miR-34a expression is related to p53 status [39]. Tan et al. compared the expression of miR-34a in osteosarcoma cell lines p53 wild-type U2OS and p53-deficient Saos2, and the results showed significantly lower expression of miR-34a in Saos2 cells. It was demonstrated that restoration of miR-34a in Saos2 cells would not increase apoptosis. miR-34a downregulates SIRT1 by elevation of NF-*κ*B levels. Adding aspirin (2 mM) to miR-34a restored Saos2 cells leading to decreased NF-*κ*B amounts and elevated apoptosis in Saos2 cells. To conclude, combination therapy with aspirin and miR-34a increased cell apoptosis in Saos2 cells [40].

### 2.2. Colorectal Cancer

Transcription factor 7 like 2 (TF7L2 or TCF4) is a transcription factor in the Wnt/*β*-catenin/TCF signaling pathway that participated in regulating several target genes [41]. Lan et al. elucidated that miR-21 has a differential expression between normal and colon cancer tissues [10]. miR-21 is a TCF4 target, and its expression is increased in various tumors [42]. Blocking the Wnt/*β*-catenin/TCF signaling pathway by aspirin (10 mM) resulted in the downregulation of miR-21 and confirmed that TCF4 could control miR-21 expression in colon carcinogenesis [10].

In a similar study on colorectal cancer, after treating the cells with aspirin (100 *μ*M), 28 lncRNAs increased that the most considerable change among them belonged to lncRNA OLA1P2. It was found that aspirin promotes the transcription of OLA1P2 by upregulating FOXD3. OLA1P2 could block phosphorylated STAT3 homodimer formation and activate the STAT3 signaling pathway, inhibiting colorectal cancer cell growth and metastasis [9].

### 2.3. Breast Cancer

Glycolysis is a critical process in cancer stem cell pathogenesis [43]. Progressive cancer cells use aerobic glycolysis rather than oxidative phosphorylation [44]. Glycolysis produces molecules, such as acetyl-CoA, to accelerate DNA replication that induces cell proliferation [45]. It has been shown that pyruvate dehydrogenase kinase 1 (PDK1) is abundant in breast cancer stem cells. Reducing PDK1 significantly diminished the ALDH^+^ subpopulation and decreased stemness-related transcriptional factor expression, sphere formation, and tumor growth. It was demonstrated that lncRNA H19 contributed to glycolysis and maintenance of breast cancer stem cells, with a trial on hypoxia-related lncRNAs [46]. H19, an endogenous RNA, could upregulate hypoxia-inducible factor 1*α* (HIF1*α*) expression by sponging let-7, which subsequently upregulates PDK1 expression. It was demonstrated that aspirin (5 mM) reduced glycolysis, glucose uptake, lactate production, ATP levels, and stem-like cancer feature by inhibiting both H19 and PDK1 in MDA-MB-231 and MCF-7 cells [46].

Bhardwaj and colleagues stated that 5′ isomiRNA from miR-140-3p (miR-140-3p-1) and its direct targets, HMG-CoA reductase (HMGCR) and HMG-CoA synthase 1 (HMGCS1), critical enzymes for the biosynthesis of cholesterol, were negatively regulated in the conversion of normal cells to preneoplastic cells [47]. It was shown that miR-140-3p-1 downregulation diminished cell growth, and this miRNA was directly linked to HMGCR and HMGCS. According to this supposition, researchers found that targeting miR-140-3p-1 and its reduction with fluvastatin (5 *μ*M) limits the preneoplastic growth of MCF10.AT1 cells and reduces the colony formation by MCF10.AT1 and MCF10.DCIS cells. They found that inhibition of cholesterol leads to the elimination of tumorigenesis. To inhibit the response of HMGCR to statins, they treated the fluvastatin-resistant preneoplastic cells with an AMP-activated protein kinase activator (AMPK) to prevent the cholesterol feedback pathway. The initiation of AMPK by aspirin (0.5 mM and one mM) strongly reduces the high-level HMGCR-induced statin. Therefore, combination therapy with fluvastatin and aspirin can prevent triple-negative breast cancer (TNBC) [47].

According to the studies, COX inhibitors can decrease the probability of breast malignancy [48]. Wong and his collaborators indicated that miR-98 and miR-222 expression was reduced in mouse breast tumor tissues after treatment with aspirin (200 ppm) and celecoxib (1500 ppm), and malignant cell growth was prevented [49].

### 2.4. Gastric Cancer

Mikami and colleagues treated tumor-bearing mice orally with 100 *μ*L aspirin (20 mg/kg of body weight) daily. The aspirin administered to the mice was similar to a human dosage of about 80–110 mg/day, showing a more remarkable decrease in microvessel density (MVD) (an indicator of tumor-associated neovascularization) than the control group. Based on the *in vitro* experiments, gastric cancer cell line, MKN-45, NUGC-3, and AGS, proliferation was increased after coincubation with platelets, suppressed by aspirin (1 mM). The findings demonstrated different expressions of miR-4670-5p in response to incubation with platelet aggregation or the addition of aspirin. Aspirin could diminish platelet-induced cancer cell proliferation, and miR-4670-5p may be an essential player in these responses [50].

miR-21 and VEGF expression was upregulated in gastric cancer in vivo and in vitro, while PPAR*α* was downregulated; expression of VEGF and PPAR*α* was correlated with miR-21 levels. Aspirin (1 mM) and apatinib (0.1 mM) for 24 hours, respectively, accelerate PPAR*α* expression and inhibit VEGFR2 phosphorylation. The activation of PPAR*α* downregulated the levels of AKT and miR-21 in GC cells. All in all, aspirin and apatinib inhibited cell proliferation and decreased migration, viability, and MKN-45 cell colony growth [8].

### 2.5. Lung Cancer

Recent research has identified that NSAIDs have suppressing effects on cigarette smoke-induced lung tumors, either mainstream (MCS) or environmental (ECS) in mice [51]. Izzotti and colleagues analyzed 1135 miRNAs in the lung and serum of mice subjected to smoke and/or oral usage of either aspirin (1600 mg/kg) or naproxen (320 mg/kg). Aspirin could regulate some miRNAs out of 1135 pulmonary miRNAs, including miR-16 in apoptosis, miR-133 in inflammation, miR-137 in cell proliferation and negative regulation of COX-2, miR-191 in COX regulation and cell proliferation, miR-199b in COX activation, miR-223 in stress response and protein repair and k-Ras regulation, and miR-543 in stress response and inflammation in pulmonary cancer [52]. Inflammatory stimulators can help lung cancer development [53]. miRNAs are new classes of inflammatory mediators that interact with inflammation and tumorigenesis [54]. Wang and coworkers found that IL-1*β* is abundant in non-small-cell lung cancer (NSCLC) patients. *In vitro* investigations demonstrated that IL-1*β* increases the growth and migration of NSCLC cell lines H460 and H1299 by downregulating miR-101, a miRNA with a tumor suppressive property, through the COX-2-HIF1*α* pathway. Lin28B, a target of miR-101, has been shown to have tumor-suppressive effects. miR-101 also upregulates the let-7 family by regulating Lin28B. IL-1*β* increases Lin28B through miR-101 downregulation. Interestingly, inhibition of COX-2 using aspirin (1 mM) and celecoxib (25 *μ*M), IL-1*β*-mediated suppression of miR-101, and IL-1*β*-mediated activation of Lin28B inhibited NSCLC cell proliferation and migration. These data show that aspirin can reverse the IL-1*β* effect on the miR-101-Lin28B-let-7 regulatory axis and antagonizes the IL-1*β* effect on NSCLC cells [55]. In a similar study, it was shown that aspirin (5 mM) significantly suppressed NSCLC cancer cell stability (A549 and H1299 cell lines) and decreased cancer cell concentration by upregulating miR-98 as a tumor suppressor and downregulating its target gene, WNT1, in lung cancer cells [56].

### 2.6. Papillary Thyroid Carcinoma

Estrogen receptor *β* (ER*β*), a key factor in thyroid malignancies [57], is upregulated in papillary thyroid carcinoma stem cells (PTCSCs), and its degradation reduces the expression of stemness-related factor ALDH^+^ cell concentrations, sphere formation, and tumor growth. lncRNA H19 was overexpressed in PTCSCs and PTC tissues by estradiol (E2) via ER*β*. The silencing of H19 can inhibit E2-induced stem-like traits. It was demonstrated that aspirin (5 mM) treatment regulates E2-induced cancer stem-like by downregulation of H19 and ER*β* expression in mice [58].

### 2.7. Hepatocellular Carcinoma

An experimental study demonstrated that treatment with doxorubicin reduced the ability to form colonies by hepatocellular side population (SP) and non-SP cells. However, the doxorubicin effect on SP cells has been more than non-SP cells. Doxorubicin inhibited SP stability, but by adding aspirin (2.5 *μ*mol/mL), the inhibitory effect of doxorubicin (500 ng/mL) significantly increased. Compared to non-SP cells, miR-491 expression in SP cells was reduced more in which aspirin had a significant effect. miR-491 directly controls ABCG2 expression. In the existence of doxorubicin and miR-491 inhibitors, aspirin's inhibition decreases the stability of SP cells, but the suppression of ABCG2 reverses it. Moreover, it was indicated that miR-130b, miR-491, miR-612, miR-3650, and miR-7-5p expressions were negatively regulated in SP cells, but aspirin only reverses the expression of miR-491. Aspirin treatment could inhibit ABCG2 expression in SP cells, which is much higher than non-SP cells. Therefore, aspirin increases SP cells' sensitivity to doxorubicin by regulating the miR-491/ABCG2 signaling pathway [59].

### 2.8. Nasopharyngeal Carcinoma

Epstein-Barr virus (EBV) expresses viral proteins in nasopharyngeal carcinoma (NPC) and large amounts of BamHI-A rightward transcripts (BARTs) that contain lncRNAs and BART miRNAs [60]. It was shown that NF-*κ*B activates BART promoters in infected cells with EBV in NPC. BART miRNAs and lncRNAs are associated with NF-*κ*B activity in infected epithelial cells during EBV harboring. NPC C666-1 cells treated with aspirin (4 mM) and NF-*κ*B kinase inhibitor, PS-1145 (0.2 mM), suppressed NF-*κ*B activity leading to a decrease in BART expression [61].

### 2.9. Coronary Artery Disease

miRNAs are responsible for the pathogenesis of several cardiovascular diseases [62]. Tang and colleagues showed that high levels of miR-142 were detected in plasma samples related to adverse cardiovascular events in coronary artery disease (CAD) patients who had undergone percutaneous coronary intervention (PCI) and administration of aspirin (200 mg) and clopidogrel (300 mg). The researchers reported that miR-142 could be a biomarker for MACE prediction in CAD patients. Additionally, miR-126, miR-130a, and miR-27 expressions increased in aspirin-sensitive and clopidogrel-resistant patients. Besides, miR-21 has downregulated in clopidogrel-resistant patients. Accordingly, these miRNAs are associated with antiplatelet therapy efficiency [63]. In another study, the correlation between miR-96-5p, miR-495-3p, miR-107, miR-223-3p, miR-15a-5p, miR-365-3p, and miR-339-3p and platelet response was investigated in 155 patients with CAD. Patients had anticoagulant therapy with aspirin (loading 300 mg, then 100 mg once daily) and clopidogrel (standard dose: loading 300 mg, then 75 mg once daily), aspirin and ticagrelor (loading 180 mg, then 90 mg twice daily), and aspirin and cilostazol (100 mg twice daily). The findings demonstrated that seven miRNAs are affected by the platelet activity level; however, the expression of miR-365-3p elucidated the most remarkable association with platelet activity, with higher expression levels correlated with higher platelet activity [62].

It was suggested that a reduction in the plasma level of miR-223, mainly from the platelet source, is an indicator of the effectiveness of antithrombic therapy [64]. However, the platelet response was correlated with a reduction in the expression of miR-223 in the plasma of CAD patients and dual antiplatelet therapy (DAPT) treatment, including low-dose aspirin (75-100 mg) and low-dose clopidogrel (300-600 mg and 75 mg). Based on the results, it was proposed that low levels of miR-223 could be considered a biomarker for platelet response to DAPT [65].

### 2.10. Platelet-Associated Cardiovascular Disease

Platelets are the main sources of circulatory miRNAs [66]. miRNAs are attractive biomarkers for monitoring multiple cardiovascular disease progression [67]. Interestingly, the levels of certain miRNAs correlate with platelet activation levels [68]. Aspirin is one of the most important antiplatelet drugs used as secondary prevention in cardiovascular disease progression [54]. However, aspirin's effectiveness can be limited since 10 to 20 percent of patients with aspirin-treated arterial thrombosis encounter a recurring vascular disorder during long-term follow-up [69].

A study on 15, 35-60-year-old healthy male volunteers without a family history of cardiovascular disease with no medication history demonstrated altered expression of six miRNAs after aspirin treatment (100 mg once daily, for two weeks), which include miR-1225-3p, miR-1271, miR1537-5p, miR-19b-1-5p, miR-548e, and miR-587. These changes were related to decreased platelet aggregation. Also, it was shown that downregulation of miR-19b-1-5p after treatment with aspirin was along with the accumulation of stable platelets in the presence of indomethacin (200 *μ*mol/L), indicating insensitivity to aspirin. Therefore, miR-19b-1-5p can be an appropriate indicator of aspirin insensitivity in patients with cardiovascular diseases [70].

Carino et al. demonstrated that the circulating levels of miR-126, miR-223, and miR-150 were remarkably decreased, while the level of miR-96 was increased after switching from aspirin (100 mg/day) and clopidogrel (75 mg/day) to ticagrelor (90 mg BD) [71]. miR-126 is associated with endothelial cell function, and angiogenesis and recent research show that this miRNA could be regarded as a biomarker in vascular disease. According to de Boer and colleagues, in pathophysiological conditions related to platelets' activation, such as type 2 diabetes, treatment with aspirin (330 *μ*mol/L) might decrease circulating miR-126 levels [72]. Overexpression of multidrug resistance protein 4 (MRP4) causes increased platelet reactivity in aspirin treatment [73]. It was demonstrated that MRP4 inhibition downregulated platelet function and increased thrombosis. There is a negative association between miR-21 and MRP4-PPAR*α* in the presence of aspirin. In megakaryoblastic cell line (DAMI), miR-21 mimic transfection decreased MRP4 and PPAR*α* mRNA expression, even if transfected cells would not be treated with aspirin. Aspirin (50 *μ*mol/L) therapy in human megakaryocytes reduced miR-21 and upregulated MRP4. miR-21 inhibited MRP4 and PPAR*α* transcription, and aspirin prevented these events [74].

Platelet reactivity is different among cardiovascular patients and has variable clinical outcomes in the patients treated with antiplatelet drugs [75]. It was shown that downregulated miR-135a-5p and miR-204-5p are related to platelet reactivity, and these miRNAs were suggested as regulatory candidates in patients with cardiovascular diseases treated with aspirin (100 mg/day). These miRNAs can have synergistic effects on seven overlapping genes (THBS1, CDC42, CORO1C, SPTBN1, TPM3, GTPBP2, and MAPRE2) [76].

MRP4 overexpression has been recently reported as a factor in reducing aspirin efficacy after bypass surgery [77]. In patients treated with aspirin (100 mg), MRP4 protein expression was upregulated, and miR-26b was decreased. Moreover, the results showed that transfecting DAMI cells with miR-26b reduced MRP4 expression in aspirin-treated cells. miR-26b has an essential effect on MRP4 modulation, and it was revealed that the incubation of platelets with this miRNA could downregulate MRP4, but it will be inhibited by aspirin treatment [78].

About 25% of cardiovascular patients deal with inadequate platelet inhibition following treatment with aspirin [79]. Aspirin resistance can be figured out using miR-92 profiling and platelet distribution width. miR-92a levels in the aspirin responders, aspirin-resistant, and control groups were investigated, and all groups showed a miR-92a downregulation after aspirin therapy (75, 100, and 150 mg per day). The findings showed that plasma miR-92a could potentially contribute to identifying aspirin resistance [80]. It was also observed that plasma levels of miR-223, miR-191, miR-126, and miR-150 decreased during platelet inhibition. These miRNAs were used as biomarkers to detect antiplatelet therapy effectiveness, which included prasugrel (10 mg), followed by a low dose of aspirin (75 mg in the second week) and higher doses of aspirin (300 mg in the third week). The results indicated that the increased aspirin dose combined with prasugrel led to increased platelet inhibition [68].

### 2.11. Parkinson's Disease

Parkinson's disease (PD) is a fatal neurologic disease with few effective treatments [81]. It was shown that miR-21, which plays a preservative role in Alzheimer's disease [82], was associated with PPAR*α* in PD. In PD patients, the level of miR-21 was increased, and PPAR*α* was reduced. DHA (1000 mM) and aspirin (1000 mM) could activate RXR*α* and PPAR*α*. Besides, DHA could increase the expression of PPAR*α* by suppressing miR-21 in SH-Y5Y cells. Combining DHA and aspirin effectively increased the heterodimer formations of PPAR*α* and RXR*α* and expression of the postsynaptic density protein 95 (PSD-95), brain-derived neurotrophic factor (BDNF), and glial cell line-derived neurotrophic factor (GDNF), whereas inhibited NF-*κ*B and COX-2. In general, the synergism of DHA and aspirin can exert neuroprotective effects through the suppression of miR-21 and activation of RXR*α* and PPAR*α* [83].

### 2.12. Preeclampsia

Preeclampsia, a disease followed by inflammation and endothelial cell disorder, is correlated with a decreased activity of endothelial nitric oxide synthase/nitric oxide (eNOS/NO) [84]. Circulating levels of proinflammatory cytokines such as tumor necrosis factor- (TNF-) *α* are increased in maternal and cord blood in patients with preeclampsia [85], leading to endothelial dysfunction via various mechanisms such as reactive oxygen species- (ROS-) mediated oxidative stress [86], which results in the progression of hypertension and proteinuria [87]. TNF-*α* and ROS activate NF-*κ*B that participated in expressing various genes associated with the pathogenesis of inflammatory diseases, including preeclampsia [88]. It was demonstrated that aspirin (5 mM) could prevent endothelial cell dysfunction and preeclampsia by preventing NF-*κ*B-dependent miR-155 and decreasing eNOS expression in human umbilical vein endothelial cells (HUVECs) [3].

### 2.13. Herpes Simplex Virus-Induced Corneal Immunopathology

Stromal keratitis (SK) is a chronic ocular lesion affected by Herpes simplex virus 1 (HSV1) infection, which is a regular etiology of vision impairment in humans [89]. Ulcers in the cornea are initially caused by neutrophils and CD4^+^ T cells in acute participation [90]. After aspirin-triggered resolvin D1 (AT-RvD1) (150 ng/eye; 5 *μ*L drop) therapy, the degree of neovascularization and stromal keratitis injuries in mice with ocular infection of HSV-1 was reduced. AT-RvD1 acts by multiple mechanisms, including suppressing proinflammatory mediators including IL1*β*, IL6, IL-12, MIP-2, MCP-2, CXCL1, VEGF, and MMP9, and also, proinflammatory miRNAs such as miR-223, miR-155, and miR-132 participated in SK and corneal neovascularization pathogenesis. Thus, AT-RvD1 treatment could be a useful strategy for managing virus-related immunopathology [91].

### 2.14. Hepatic Ischemia

Liver ischemia/reperfusion (I/R) is a critical morbidity factor associated with several clinical outcomes, such as hepatectomy, liver transplantation, and trauma. In such situations, the accumulation of inflammatory cells and mediators, ROS, and further biochemical imbalance in intracellular homeostasis lead to hepatocellular damage after I/R [92]. Inflammation has an essential role in tissue damage throughout liver ischemia [93]. Resolvin D1 (RvD1) is a pivotal factor in reducing liver damage by inhibiting inflammatory responses [94].

AT-RvD1 is a member of specialized proresolving lipid mediators (SPMs) and is biosynthesized by an omega-3 fatty acid (DHA) and has been shown to promote resolution in many inflammatory diseases [95, 96]. AT-RvD1, the 17R epimer of RvD1, is more durable and resistant to catalysis than RvD1 [97]. AT-RvD1 begins resolution pathways by attaching to the high-affinity G protein-coupled receptors (GPCRs), containing the LXA4 receptor (ALX/FPR2) and GPR32 [96], and downregulation of TNF-*α* stimulated NF-*κ*B [98]. Both RvD1 and AT-RvD1 are potential compounds for treating several human inflammation diseases, including inflammatory pain [99, 100], arthritis [101], peritonitis [102], kidney ischemia/reperfusion injury [103], and sepsis [104]. It was indicated that the usage of RvD1 before hepatic I/R alleviates hepatic damage through suppression of inflammatory responses [105]. Besides, it was shown that during self-limited acute inflammatory, RvD1 upregulated miRNA-146b [106], which inhibited the expression of TNF receptor-associated factor 6 (TRAF6) in human umbilical vein endothelial cells [107].

TRAF6, as a target of miR-146b, involves NF-*κ*B activation [108, 109]. Treatment with AT-RvD1 (5 *μ*g/kg) in an animal model of liver ischemia remarkably downregulated alanine aminotransferase (ALT), aspartate aminotransferase (AST), and liver tissue damage. Additionally, AT-RvD1 considerably suppressed inflammatory responses, as demonstrated by ameliorating TNF*α* and myeloperoxidase and apoptosis inhibition. Moreover, AT-RvD1 pretreatment upregulated the expression of miR-146b in the liver of the rats with hepatic impairment. Downregulation of miR-146b suppressed TRAF6 and NF-*κ*B expression in the liver. Therefore, AT-RvD1 treatment alleviates hepatic injury by modulating miR-146b [110].

### 2.15. Radiation Therapy

Radiation harms the heart during cancer therapy, mainly due to oxidation and inflammation [111]. Viczenczova and coworkers showed that a separate dose of radiation could increase connexin 43 (Cx43) in the myocardium, activate protein kinase C (PKC) signaling through miR-1 downregulation, and miR-21 (with a role in myocardial remodeling and apoptosis) upregulation in the left ventricle of male rats. Also, it was demonstrated that antioxidant and anti-inflammatory drugs with vasodilating properties such as aspirin (3 mg/day) and atorvastatin (0.25 mg/day) could increase myocardial response in the left and right ventricles during radiation. Aspirin treatment prevented the upregulation of Cx43 (allows electrical connection and intercellular interconnection) and PKC*ε* expression with no changes in miR-1 levels. Also, this treatment prevented miR-21 upregulation in the left ventricle, which was associated with improved radiation-induced changes in the Cx43 myocardium protein and miR-21, possibly due to the improvement of oxidative stress and inflammation [112].

## 3. Conclusion and Perspectives

Aspirin is one of the most famous ancient drugs that has been used in human and nonhuman studies as a therapeutic agent in various diseases. On the other hand, numerous studies have shown the role of different ncRNAs as diagnostic, prognostic, and therapeutic molecules. Because of the importance of both, we conducted a study to evaluate the effects of aspirin on the expression of ncRNAs through a mechanistic approach.

Effects of aspirin alone or in combination with other medications such as statins, P2Y2 antagonists, and tyrosine kinase inhibitors on ncRNAs affect different cellular and molecular pathways. In different disease models, various ncRNAs and their effects on cellular pathways were affected by aspirin, of which miRNAs including miR-155, miR-21, miR-98, miR-191, miR-126, miR-223, and miR-150 and lncRNA H19 were the most common. Elucidating the molecular networks of the ncRNAs related to aspirin and their impacts on cellular functions will help better understand its mechanistic diversity as one of the most widely used drugs. The effects of aspirin on the expression of different ncRNAs in various diseases are more investigated than the other NSAIDs including celecoxib [37] and ibuprofen [113]; however, further investigations are recommended to evaluate aspirin effects on diseases through the expression of ncRNAs.

## Figures and Tables

**Table 1:** Classification of ncRNAs.

ncRNA type	Abbreviation	Full name	Function	Nucleotides	References
Housekeeping	rRNAs	Ribosomal RNAs	Ribosomal component during translation	7216	[24]
	tRNAs	Transfer RNAs	Adaptor in translation	76-90	[24]
	snRNAs	Small nuclear RNAs	RNA splicing	60-300	[114, 115]
	tel-sRNAs	Telomere small RNAs	Telomere maintenance	24	[24, 116]
	snoRNAs	Small nucleolar RNAs	Chemical modifications (methylation and pseudouridylation) of other ncRNAs (rRNA, tRNA, snRNA); alternative splicing; cis- and trans-gene regulation; may also function as miRNA	70-200	[24]
Regulatory	miRNAs	MicroRNAs	Gene silencing: translational repression or RNA degradation	18-15	[117, 118]
	siRNAs	Small interfering RNAs	Gene regulation, transposon control, and viral defense	21-23	[118, 119]
	piRNAs	PiwiRNAs	Transposon repression, chromatin modification	24-30	[118, 120, 121]
	eRNAs	Enhancer derived RNAs	Regulation of gene expression	50-2000	[24]
	LncRNAs	Long noncoding RNAs	Gene regulatory processes: promoter-specific repression, activation of epigenetic gene regulation	200-100,000	[117, 122, 123]
	CircRNAs	Circular RNAs	Serving as RNA sponges (ceRNAs) to bind miRNAs and modulate miRNA-targeted gene expression	>200	[124]
	xiRNAs	X-inactivation RNAs	X-chromosome inactivation in placental mammals	>200	[125, 126]
	gRNAs	Guide RNAs	RNA editing	100	[24]
	Promoter-associated RNAs (PARs)		A general term encompassing a suite of long and short RNAs, including promoter-associated RNAs (PASRs) and transcription initiation RNAs (tiRNAs) that overlap promoters and TSSs. These transcripts may regulate gene expression	20-200	[127, 128]
	Sno-derived RNAs	sdRNAs	Small RNAs, some of which are Dicer-dependent, which are processed from small nucleolar RNAs (snoRNAs). Some sdRNAs have been shown to function as miRNA-like regulators of translation	20-24	[129, 130]
	MicroRNA-offset RNAs	moRNAs	Small RNAs, derived from the regions adjacent to pre-miRNAs. Their function is unknown	20	[131, 132]
	tRNA-derived RNAs		tRNAs can be processed into small RNA species by a conserved RNase (angiogenin). They are able to induce translational repression	73-90	[133]
	MSY2-associated RNAs	MSY-RNAs	MSY-RNAs are associated with the germ cell-specific DNA/RNA binding protein MSY2. Like piRNAs, they are largely restricted to the germline. Their function is unknown	26–30	[134]
	Centrosome-associated RNAs	crasiRNAs	Derived from centrosomes for local chromatin modification	34–42	[135]

**Table 2:** Effects and consequences of aspirin on ncRNAs in different conditions.

Treatment	Cell type	Effects on ncRNAs	Outcomes	References
Aspirin (5 mM)	Human umbilical vein endothelial cells) HUVECs(	Downregulation of miR-155	Downregulation of eNOS and NF-*κ*B	[3]
Aspirin (1 mM)	MKN-45 cells (gastric cancer cell line)	Downregulation of miR-21	Upregulation of PPAR*α*, downregulation of VEGFR2 phosphorylation and AKT	[8]
Aspirin (1 mM)+apatinib (0.1 mM)	MKN-45 cells (gastric cancer cell line)	Downregulation of miR-21	Upregulation of PPAR*α*, downregulation of VEGFR2 phosphorylation, AKT, migration and colony formation	[8]
Aspirin (100 *μ*M)	Colorectal cancer cells (primary and cell lines)	Upregulation of lncRNA OLA1P2	Upregulation of FOXD3, activating STAT3 pathway	[9]
Aspirin (10 mM)	LS174T cells (colorectal cancer cell line)	Downregulation of miR-21	Downregulation of Wnt/*β*-catenin/TCF4 signaling	[10]
Aspirin (5 mM)	MDA-MB-231, MCF-7, SK-BR-3, and HEK293T cells (breast cancer cell lines)	Downregulation of lncRNA H19	Downregulation of PDK1, glycolysis, glucose uptake, lactate production, ATP levels and stem-like cancer characteristics	[46]
Aspirin (0.5 and 1 mM)+fluvastatin (5 *μ*M)	MCF10.AT1 and MCF10.DCIS cells (MCF10A-based model for breast cancer)	Upregulation of miR-140-3p-1	Downregulation of HMGCR and HMGCS1	[47]
Aspirin (200 ppm)	MCF-7 (breast cancer cells)	Upregulation of miR-222, miR-98, and miR-145	Downregulation of Bcl-xl	[49]
Aspirin (100 *μ*L)	MKN-45 cells (gastric cancer cell line)	Downregulation of miR-4670-5P	—	[50]
Aspirin (1600 mg/kg)	Lung of mice	Downregulation of miR-30e, miR-32, miR-380, and miR-543	Downregulation of proliferation by non-prostaglandin-dependent pathways	[52]
Aspirin (1600 mg/kg)	Serum of mice	Downregulation of miR-16, miR-133, miR-137, and miR-191	Downregulation of proliferation affects non-prostaglandin-dependent pathways	[52]
Aspirin (1600 mg/kg)	Lung and serum of mice	Downregulation of miR-30c, miR-181b, miR-183, miR-301a, miR-350, miR-466a-3p, miR-466i-3p, miR-500, and miR-709	Downregulation of proliferation by non-prostaglandin-dependent pathways	[52]
Aspirin (1 mM)	Human NSCLC cell lines H460 and H1299 cell line	Upregulation of miR-101 and let-7 miRNA family	COX-2, IL-1*β*-mediated repression of miR-101, IL-1*β*-mediated activation of Lin28B, cell proliferation, and migration	[55]
Aspirin (2.5 mM and 5 mM)	A549 and H1299 lung cancer cell lines	Upregulation of miR-98	Downregulation of WNT1	[56]
Aspirin (5 mM)	Human thyroid cancer cell lines (TPC-1 and K-1)	Downregulation of lncRNA H19	Downregulation of estradiol and ER*β*	[58]
Aspirin (2.5 *μ*mol/mL)	Non-SP and SP cells isolated from MHCC-97L cell line	Upregulation of miR-491	Downregulation of ABCG2 protein expression	[59]
Aspirin (4 mM)	C666-1 cell line (nasopharyngeal carcinoma cells)	Downregulation of BART miRNAs	Downregulation of NF-*κ*B activity	[61]
Aspirin (300 mg and 100 mg)+clopidogrel (300 mg and 75 mg) Aspirin+ticagrelor (180 mg and 90 mg) Aspirin+clopidogrel+cilostazol (100 mg)	Platelet-rich plasma of CAD patients	Upregulation of miR-365-3p	Upregulation of platelet activity	[62]
Aspirin (100 mg)+clopidogrel (300 mg)	Blood samples of CAD patients	Upregulation of miR-126, miR-130a, miR-142, and miR-27	—	[63]
Aspirin low dose (75-100 mg)+clopidogrel (300–600 and 75 mg)	Plasma CAD patients	Downregulation of miR-223	—	[65]
Aspirin (75 and 300 mg)+prasugrel (10 mg)	Platelets of patients	Downregulation of miR-223, miR-191, miR-126, and miR-150	Downregulation of platelet	[68]
Aspirin (100 mg)+indomethacin (200 *μ*mol/L)	Platelets of healthy males	Downregulation of miR-19b-1-5p	—	[70]
Aspirin (100 mg/day)+clopidogrel (75 mg/day)+ticagrelor (90 mg/BD)	Plasma of patients	Downregulation of miR-126, miR-150, and miR-223, upregulation of and miR-96	—	[71]
Aspirin (330 *μ*mol/L)	Plasma or platelet of healthy volunteers	Downregulation of miR-126	—	[72]
Aspirin (100 and 300 mg/day, 50 *μ*mol/L)	Human platelets and DAMI cells (human megakaryoblastic)	Downregulation of miR-21	Upregulation of MRP4 and PPAR*α*	[74]
Aspirin (100 mg/day)	Platelets of atherothrombotic patients	Downregulation of miR-135a-5p and miR-204-5p	—	[76]
Aspirin (100 mg/day)	Platelets of patients	Downregulation of miR-26b	Upregulation of MRP4	[78]
Aspirin (75 and 100 mg, 150 mg/day)	Plasma of patients	Downregulation of miR-92a	Aspirin resistance	[80]
Aspirin (1000 mM)+DHA (1000 mM)	SH-Y5Y cell line	Downregulation of miR-21	Upregulation of PPAR*α* and RXRa, PSD-95, BDNF, GDNF, downregulation of NF-*κ*B and COX-2	[83]
AT-RvD1 (150 ng/eye; 5 *μ*L drop)	Corneal cells of mice	Downregulation of miR-223, miR-155, and miR-132	Downregulation of proinflammatory mediators such as IL1*β*, IL6, and IL-12, as well as MIP-2, MCP-2, CXCL1, VEGF, and MMP9	[91]
AT-RvD1 (5 *μ*g/kg)	Liver of rats	Upregulation of miR-146b	Downregulation of TRAF6 and NF-*κ*B, ALT, AST, and liver tissue damage, amelioration of TNF*α* and myeloperoxidase	[110]
Aspirin (3 mg/day)+atorvastatin (0.25 mg/day)	Myocardial cells of rats	Downregulation of miR-21	Improvement of radiation-induced changes in the Cx43, improvement of oxidative stress and inflammation	[112]

## References

[1] Du G., Lin Q., Wang J. (2016). A brief review on the mechanisms of aspirin resistance. *International Journal of Cardiology*.

[2] Patrignani P., Patrono C. (2016). Aspirin and cancer. *Journal of the American College of Cardiology*.

[3] Kim J., Lee K. S., Kim J. H. (2017). Aspirin prevents TNF-*α*-induced endothelial cell dysfunction by regulating the NF-*κ*B-dependent miR-155/eNOS pathway: role of a miR-155/eNOS axis in preeclampsia. *Free Radical Biology and Medicine*.

[4] Gala M. K., Chan A. T. (2015). Molecular pathways: aspirin and Wnt signaling-a molecularly targeted approach to cancer prevention and treatment. *Clinical Cancer Research*.

[5] Nicolas F. E. (2017). Role of ncRNAs in development, diagnosis and treatment of human cancer. *Recent Patents on Anti-Cancer Drug Discovery*.

[6] Akbari Kordkheyli V., Khonakdar Tarsi A., Mishan M. A. (2019). Effects of quercetin on microRNAs: a mechanistic review. *Journal of Cellular Biochemistry*.

[7] Mishan M. A., Khazeei Tabari M. A., Mahrooz A., Bagheri A. (2021). Role of microRNAs in the anticancer effects of the flavonoid luteolin: a systematic review. *European Journal of Cancer Prevention*.

[8] Zhang W., Tan Y., Ma H. (2017). Combined aspirin and apatinib treatment suppresses gastric cancer cell proliferation. *Oncology Letters*.

[9] Guo H., Liu J., Ben Q. (2016). The aspirin-induced long non-coding RNA OLA1P2 blocks phosphorylated STAT3 homodimer formation. *Genome Biology*.

[10] Lan F., Yue X., Han L. (2012). Genome-wide identification of TCF7L2/TCF4 target miRNAs reveals a role for miR-21 in Wnt-driven epithelial cancer. *International Journal of Oncology*.

[11] Paseban M., Marjaneh R. M., Banach M., Riahi M. M., Bo S., Sahebkar A. (2020). Modulation of microRNAs by aspirin in cardiovascular disease. *Trends in Cardiovascular Medicine*.

[12] Yiannakopoulou E. (2014). Targeting epigenetic mechanisms and microRNAs by aspirin and other non steroidal anti-inflammatory agents--implications for cancer treatment and chemoprevention. *Cellular Oncology (Dordrecht)*.

[13] Lichtenberger L. M., Phan T., Fang D. (2016). Bioavailability of Aspirin in Rats Comparing the Drug's Uptake into GI Tissue and Vascular and Lymphatic Systems: Implications on Aspirin's Chemopreventive Action. *Journal of Physiology and Pharmacology*.

[14] Arif H., Aggarwal S. (2019). *Salicylic acid (aspirin)*.

[15] Zhang H., Guo C., Zhang A. (2012). Effect of S-aspirin, a novel hydrogen-sulfide-releasing aspirin (ACS14), on atherosclerosis in apoE-deficient mice. *European Journal of Pharmacology*.

[16] Vlachojannis J., Magora F., Chrubasik S. (2011). Willow species and aspirin: different mechanism of actions. *Phytotherapy Research*.

[17] Scheiman J. M. (2012). Prevention of damage induced by aspirin in the GI tract. *Best Practice & Research Clinical Gastroenterology*.

[18] Hobl E. L., Schmid R. W., Stimpfl T., Ebner J., Jilma B. (2015). Absorption kinetics of low-dose chewable aspirin–implications for acute coronary syndromes. *European Journal of Clinical Investigation*.

[19] Bastaki S. M., Padol I. T., Amir N., Hunt R. H. (2018). Effect of aspirin and ibuprofen either alone or in combination on gastric mucosa and bleeding time and on serum prostaglandin E 2 and thromboxane A 2 levels in the anaesthetized rats in vivo. *Molecular and Cellular Biochemistry*.

[20] Todoric J., Antonucci L., Karin M. (2016). Targeting inflammation in cancer prevention and therapy. *Cancer Prevention Research*.

[21] Jörgensen T. G., Weis-Fogh U. S., Nielsen H. H., Olesen H. (1976). Salicylate-and aspirin-induced uncoupling of oxidative phosphorylation in mitochondria isolated from the mucosal membrane of the stomach. *Scandinavian Journal of Clinical and Laboratory Investigation*.

[22] Huo X., Zhang X., Yu C. (2018). Aspirin prevents NF-*κ*B activation and CDX2 expression stimulated by acid and bile salts in oesophageal squamous cells of patients with Barrett's oesophagus. *Gut*.

[23] Long Y., Wang X., Youmans D. T., Cech T. R. (2017). How do lncRNAs regulate transcription?. *Science Advances*.

[24] Song E. (2016). *The Long and Short Non-Coding RNAs in Cancer Biology*.

[25] Ayers D., Scerri C. (2018). *Non-coding RNA influences in dementia*.

[26] Mishan M. A., Tabari M. A. K., Parnian J., Fallahi J., Mahrooz A., Bagheri A. (2020). Functional mechanisms ofmiR‐192 family in cancer. *Genes, Chromosomes and Cancer*.

[27] Huang B., Zhang R. (2014). Regulatory non-coding RNAs: revolutionizing the RNA world. *Molecular Biology Reports*.

[28] Ghalehnoei H., Bagheri A., Fakhar M., Mishan M. A. (2020). Circulatory microRNAs: promising non-invasive prognostic and diagnostic biomarkers for parasitic infections. *European Journal of Clinical Microbiology & Infectious Diseases*.

[29] Dykes I., Emanueli C. (2017). Transcriptional and post-transcriptional gene regulation by long non-coding RNA. *Genomics, Proteomics & Bioinformatics*.

[30] Toden S., Zumwalt T. J., Goel A. (2021). Non-coding RNAs and potential therapeutic targeting in cancer. *Biochimica Et Biophysica Acta. Reviews on Cancer*.

[31] Cheng J. T., Wang L., Wang H. (2019). Insights into biological role of LncRNAs in epithelial-mesenchymal transition. *Cell*.

[32] Yamada A., Yu P., Lin W., Okugawa Y., Boland C. R., Goel A. (2018). A RNA-sequencing approach for the identification of novel long non-coding RNA biomarkers in colorectal cancer. *Scientific Reports*.

[33] Xue M., Zhuo Y., Shan B. (2017). MicroRNAs, long noncoding RNAs, and their functions in human disease. *Bioinformatics in MicroRNA Research*.

[34] Mishra S., Verma S. S., Rai V. (2019). Long non-coding RNAs are emerging targets of phytochemicals for cancer and other chronic diseases. *Cellular and Molecular Life Sciences*.

[35] Delás M. J., Hannon G. J. (2017). lncRNAs in development and disease: from functions to mechanisms. *Open Biology*.

[36] Mahrooz A., Mackness M., Bagheri A., Ghaffari-Cherati M., Masoumi P. (2019). The epigenetic regulation of paraoxonase 1 (PON1) as an important enzyme in HDL function: the missing link between environmental and genetic regulation. *Clinical Biochemistry*.

[37] Mishan M. A., Tabari M. A. K., Zargari M., Bagheri A. (2020). MicroRNAs in the anticancer effects of celecoxib: a systematic review. *European Journal of Pharmacology*.

[38] Kordkheyli V. A., Mishan M. A., Tarsi A. K. (2021). MicroRNAs may provide new strategies in the treatment and diagnosis of diabetic retinopathy: importance of VEGF. *Iranian Journal of Basic Medical Sciences*.

[39] Park S. M., Gaur A. B., Lengyel E., Peter M. E. (2008). The miR-200 family determines the epithelial phenotype of cancer cells by targeting the E-cadherin repressors ZEB1 and ZEB2. *Genes & Development*.

[40] Tan J., Fan L., Mao J. J. (2012). Restoration of miR-34a in p53 deficient cells unexpectedly promotes the cell survival by increasing NF*κ*B activity. *Journal of Cellular Biochemistry*.

[41] Hou N., Ye B., Li X. (2016). Transcription factor 7-like 2 mediates canonical Wnt/*β*-Catenin signaling and c-Myc upregulation in heart failure. *Circulation. Heart Failure*.

[42] Liu X. G., Zhu W. Y., Huang Y. Y. (2012). High expression of serum miR-21 and tumor miR-200c associated with poor prognosis in patients with lung cancer. *Medical Oncology*.

[43] Ma Z., Cui X., Lu L. (2019). Exosomes from glioma cells induce a tumor-like phenotype in mesenchymal stem cells by activating glycolysis. *Stem Cell Research & Therapy*.

[44] Chae Y. C., Kim J. H. (2018). Cancer stem cell metabolism: target for cancer therapy. *BMB Reports*.

[45] Fan C., Tang Y., Wang J. (2017). Role of long non-coding RNAs in glucose metabolism in cancer. *Molecular Cancer*.

[46] Peng F., Wang J. H., Fan W. J. (2018). Glycolysis gatekeeper PDK1 reprograms breast cancer stem cells under hypoxia. *Oncogene*.

[47] Bhardwaj A., Singh H., Trinidad C. M., Albarracin C. T., Hunt K. K., Bedrosian I. (2018). The isomiR-140-3p-regulated mevalonic acid pathway as a potential target for prevention of triple negative breast cancer. *Breast Cancer Research*.

[48] Regulski M., Regulska K., Prukala W., Piotrowska H., Stanisz B., Murias M. (2016). COX-2 inhibitors: a novel strategy in the management of breast cancer. *Drug Discovery Today*.

[49] Wong T. Y., Li F., Lin S. M., Chan F. L., Chen S., Leung L. K. (2014). Celecoxib increases miR-222 while deterring aromatase-expressing breast tumor growth in mice. *BMC Cancer*.

[50] Mikami J., Kurokawa Y., Takahashi T. (2016). Antitumor effect of antiplatelet agents in gastric cancer cells: an in vivo and in vitro study. *Gastric Cancer*.

[51] Iimura Y., Shimomura H., Yasu T. (2018). NSAIDs may prevent EGFR-TKI-related skin rash in non-small cell lung cancer patients. *International Journal of Clinical Pharmacology and Therapeutics*.

[52] Izzotti A., Balansky R., Ganchev G. (2017). Early and late effects of aspirin and naproxen on microRNAs in the lung and blood of mice, either unexposed or exposed to cigarette smoke. *Oncotarget*.

[53] Grivennikov S. I., Greten F. R., Karin M. (2010). Immunity, inflammation, and cancer. *Cell*.

[54] Zhang L., Fan X. M. (2015). The pathological role of microRNAs and inflammation in colon carcinogenesis. *Clinics and Research in Hepatology and Gastroenterology*.

[55] Wang L., Zhang L. F., Wu J. (2014). IL-1*β*-Mediated repression of microRNA-101 is crucial for inflammation-promoted lung tumorigenesis. *Cancer Research*.

[56] Gan H., Lin L., Hu N. (2019). Aspirin ameliorates lung cancer by targeting the miR-98/WNT1 axis. *Thorac Cancer*.

[57] Chen G. G., Vlantis A. C., Zeng Q., van Hasselt C. A. (2008). Regulation of cell growth by estrogen signaling and potential targets in thyroid cancer. *Current Cancer Drug Targets*.

[58] Li M., Chai H. F., Peng F. (2018). Estrogen receptor *β* upregulated by lncRNA- _H19_ to promote cancer stem-like properties in papillary thyroid carcinoma. *Cell Death & Disease*.

[59] Xie Z. Y., Liu M. S., Zhang C., Cai P. C., Xiao Z. H., Wang F. F. (2018). Aspirin enhances the sensitivity of hepatocellular carcinoma side population cells to doxorubicin via miR-491/ABCG2. *Bioscience Reports*.

[60] Takada K. (2012). Role of EBER and BARF1 in nasopharyngeal carcinoma (NPC) tumorigenesis. *Seminars in Cancer Biology*.

[61] Verhoeven R. J., Tong S., Zhang G. (2016). NF-*κ*B signaling regulates expression of Epstein-Barr virus BART microRNAs and long noncoding RNAs in nasopharyngeal carcinoma. *Journal of Virology*.

[62] Chen Y. C., Lin F. Y., Lin Y. W. (2019). Platelet microRNA 365-3p expression correlates with high on-treatment platelet reactivity in coronary artery disease patients. *Cardiovascular Drugs and Therapy*.

[63] Tang Q. J., Lei H. P., Wu H. (2019). Plasma miR-142 predicts major adverse cardiovascular events as an intermediate biomarker of dual antiplatelet therapy. *Acta Pharmacologica Sinica*.

[64] Shi R., Zhou X., Ji W. J. (2015). The emerging role of miR-223 in platelet reactivity: implications in antiplatelet therapy. *BioMed Research International*.

[65] Chyrchel B., Totoń-Zurańska J., Kruszelnicka O. (2015). Association of plasma miR-223 and platelet reactivity in patients with coronary artery disease on dual antiplatelet therapy: a preliminary report. *Platelets*.

[66] Lazar S., Goldfinger L. E. (2018). Platelet microparticles and miRNA transfer in cancer progression: many targets, modes of action, and effects across cancer stages. *Frontiers in Cardiovascular Medicine*.

[67] Dhingra R., Vasan R. S. (2017). Biomarkers in cardiovascular disease: statistical assessment and section on key novel heart failure biomarkers. *Trends in Cardiovascular Medicine*.

[68] Willeit P., Zampetaki A., Dudek K. (2013). Circulating microRNAs as novel biomarkers for platelet activation. *Circulation Research*.

[69] Eikelboom J. W., Hirsh J., Weitz J. I., Johnston M., Yi Q., Yusuf S. (2002). Aspirin-resistant thromboxane biosynthesis and the risk of myocardial infarction, stroke, or cardiovascular death in patients at high risk for cardiovascular events. *Circulation*.

[70] Kok M. G. M., Mandolini C., Moerland P. D. (2016). Low miR-19b-1-5p expression in isolated platelets after aspirin use is related to aspirin insensitivity. *International Journal of Cardiology*.

[71] Carino A., De Rosa S., Sorrentino S. (2016). Modulation of circulating microRNAs levels during the switch from clopidogrel to ticagrelor. *BioMed Research International*.

[72] de Boer H. C., van Solingen C., Prins J. (2013). Aspirin treatment hampers the use of plasma microRNA-126 as a biomarker for the progression of vascular disease. *European Heart Journal*.

[73] Floyd C. N., Ferro A. (2014). Mechanisms of aspirin resistance. *Pharmacology & Therapeutics*.

[74] Massimi I., Alemanno L., Guarino M. L. (2018). miR-21 role in aspirin-dependent PPAR*α* and multidrug resistance protein 4 upregulation. *Res Pract Thromb Haemost*.

[75] Gurbel P. A., Bliden K. P., DiChiara J. (2007). Evaluation of dose-related effects of aspirin on platelet function: results from the Aspirin-Induced Platelet Effect (ASPECT) study. *Circulation*.

[76] Zufferey A., Ibberson M., Reny J. L. (2016). New molecular insights into modulation of platelet reactivity in aspirin-treated patients using a network-based approach. *Human Genetics*.

[77] Schuetz J. D., Connelly M. C., Sun D. (1999). MRP4: a previously unidentified factor in resistance to nucleoside-based antiviral drugs. *Nature Medicine*.

[78] La Rosa G., Biasucci L. M., Mandolini C. (2018). Platelet miRNA-26b down-regulates multidrug resistance protein 4 in patients on chronic aspirin treatment. *Journal of Cardiovascular Medicine (Hagerstown, Md.)*.

[79] Lordkipanidzé M. (2012). Advances in monitoring of aspirin therapy. *Platelets*.

[80] Binderup H. G., Houlind K., Madsen J. S., Brasen C. L. (2016). Aspirin resistance may be identified by miR-92a in plasma combined with platelet distribution width. *Clinical Biochemistry*.

[81] Dietrichs E., Odin P. (2017). Algorithms for the treatment of motor problems in Parkinson's disease. *Acta Neurologica Scandinavica*.

[82] Feng M. G., Liu C. F., Chen L. (2018). MiR-21 attenuates apoptosis-triggered by amyloid-*β* via modulating PDCD4/ PI3K/AKT/GSK-3*β* pathway in SH-SY5Y cells. *Biomedicine & Pharmacotherapy*.

[83] Fu Y., Zhen J., Lu Z. (2017). Synergetic neuroprotective effect of docosahexaenoic acid and aspirin in SH-Y5Y by inhibiting miR-21 and activating RXR*α* and PPAR*α*. *DNA and Cell Biology*.

[84] Matsubara K., Higaki T., Matsubara Y., Nawa A. (2015). Nitric oxide and reactive oxygen species in the pathogenesis of preeclampsia. *International Journal of Molecular Sciences*.

[85] Conrad K. P., Miles T. M., Benyo D. F. (1998). Circulating levels of immunoreactive cytokines in women with preeclampsia. *American Journal of Reproductive Immunology*.

[86] Gilbert J. S., Ryan M. J., LaMarca B. B., Sedeek M., Murphy S. R., Granger J. P. (2008). Pathophysiology of hypertension during preeclampsia: linking placental ischemia with endothelial dysfunction. *American Journal of Physiology. Heart and Circulatory Physiology*.

[87] Xie C., Yao M. Z., Liu J. B., Xiong L. K. (2011). A meta-analysis of tumor necrosis factor-alpha, interleukin-6, and interleukin-10 in preeclampsia. *Cytokine*.

[88] Vaughan J. E., Walsh S. W. (2012). Activation of NF-*κ*B in placentas of women with preeclampsia. *Hypertension in Pregnancy*.

[89] Jiang Y., Yin X., Stuart P. M., Leib D. A. (2015). Dendritic cell autophagy contributes to herpes simplex virus-driven stromal keratitis and immunopathology. *MBio*.

[90] Rowe A. M., St Leger A. J., Jeon S., Dhaliwal D. K., Knickelbein J. E., Hendricks R. L. (2013). Herpes keratitis. *Progress in Retinal and Eye Research*.

[91] Rajasagi N. K., Bhela S., Varanasi S. K., Rouse B. T. (2017). Frontline science: aspirin-triggered resolvin D1 controls herpes simplex virus-induced corneal immunopathology. *Journal of Leukocyte Biology*.

[92] Montalvo-Jave E. E., Escalante-Tattersfield T., Ortega-Salgado J. A., Pina E., Geller D. A. (2008). Factors in the pathophysiology of the liver ischemia-reperfusion injury. *The Journal of Surgical Research*.

[93] Kan C., Ungelenk L., Lupp A., Dirsch O., Dahmen U. (2018). Ischemia-reperfusion injury in aged livers-the energy metabolism, inflammatory response, and autophagy. *Transplantation*.

[94] Kang J. W., Choi H. S., Lee S. M. (2018). Resolvin D1 attenuates liver ischaemia/reperfusion injury through modulating thioredoxin 2-mediated mitochondrial quality control. *British Journal of Pharmacology*.

[95] Serhan C. N., Chiang N., Van Dyke T. E. (2008). Resolving inflammation: dual anti-inflammatory and pro-resolution lipid mediators. *Nature Reviews. Immunology*.

[96] Recchiuti A. (2013). Resolvin D1 and its GPCRs in resolution circuits of inflammation. *Prostaglandins & Other Lipid Mediators*.

[97] Sun Y. P., Oh S. F., Uddin J. (2007). Resolvin D1 and Its Aspirin-triggered 17 _R_ Epimer :. *The Journal of Biological Chemistry*.

[98] Krishnamoorthy S., Recchiuti A., Chiang N. (2010). Resolvin D1 binds human phagocytes with evidence for proresolving receptors. *Proceedings of the National Academy of Sciences of the United States of America*.

[99] Xu Z. Z., Zhang L., Liu T. (2010). Resolvins RvE1 and RvD1 attenuate inflammatory pain via central and peripheral actions. *Nature Medicine*.

[100] Bang S., Yoo S., Yang T. J., Cho H., Hwang S. W. (2012). 17(R)-Resolvin D1 specifically inhibits transient receptor potential ion channel vanilloid 3 leading to peripheral antinociception. *British Journal of Pharmacology*.

[101] Lima-Garcia J. F., Dutra R. C., da Silva K., Motta E. M., Campos M. M., Calixto J. B. (2011). The precursor of resolvin D series and aspirin-triggered resolvin D1 display anti-hyperalgesic properties in adjuvant-induced arthritis in rats. *British Journal of Pharmacology*.

[102] Tang Y., Zhang M. J., Hellmann J., Kosuri M., Bhatnagar A., Spite M. (2013). Proresolution therapy for the treatment of delayed healing of diabetic wounds. *Diabetes*.

[103] Duffield J. S., Hong S., Vaidya V. S. (2006). Resolvin D series and protectin D1 mitigate acute kidney injury. *Journal of Immunology*.

[104] Chen F., Fan X. H., Wu Y. P. (2014). Resolvin D1 improves survival in experimental sepsis through reducing bacterial load and preventing excessive activation of inflammatory response. *European Journal of Clinical Microbiology & Infectious Diseases*.

[105] Zhang T., Shu H. H., Chang L., Ye F., Xu K. Q., Huang W. Q. (2015). Resolvin D1 protects against hepatic ischemia/reperfusion injury in rats. *International Immunopharmacology*.

[106] Recchiuti A., Krishnamoorthy S., Fredman G., Chiang N., Serhan C. N. (2011). MicroRNAs in resolution of acute inflammation: identification of novel resolvin D1-miRNA circuits. *The FASEB Journal*.

[107] Perry M. M., Williams A. E., Tsitsiou E., Larner-Svensson H. M., Lindsay M. A. (2009). Divergent intracellular pathways regulate interleukin-1beta-induced miR-146a and miR-146b expression and chemokine release in human alveolar epithelial cells. *FEBS Letters*.

[108] Shimo Y., Yanai H., Ohshima D. (2011). TRAF6 directs commitment to regulatory T cells in thymocytes. *Genes to Cells*.

[109] Miyata R., Kakuki T., Nomura K. (2015). Poly(I:C) induced microRNA-146a regulates epithelial barrier and secretion of proinflammatory cytokines in human nasal epithelial cells. *European Journal of Pharmacology*.

[110] Zhang T., Xiu H. H., Liu J. X., Ma Y., Xu K. Q., Huang W. Q. (2017). Protective effect of aspirin-triggered resolvin D1 on hepatic ischemia/reperfusion injury in rats: the role of miR-146b. *International Immunopharmacology*.

[111] Puukila S., Lemon J. A., Lees S. J., Tai T. C., Boreham D. R., Khaper N. (2017). Impact of ionizing radiation on the cardiovascular system: a review. *Radiation Research*.

[112] Viczenczova C., Kura B., Egan Benova T. (2018). Irradiation-induced cardiac connexin-43 and miR-21 responses are hampered by treatment with atorvastatin and aspirin. *International Journal of Molecular Sciences*.

[113] El-Lithy G. M., El-Bakly W. M., Matboli M., Abd-Alkhalek H. A., Masoud S. I., Hamza M. (2016). Prophylactic L-arginine and ibuprofen delay the development of tactile allodynia and suppress spinal miR-155 in a rat model of diabetic neuropathy. *Translational Research*.

[114] Eddy S. R. (2001). Non-coding RNA genes and the modern RNA world. *Nature Reviews Genetics*.

[115] Makunin J. M. I. (2006). Non-coding RNA. *Human Molecular Genetics*.

[116] Cao F., Li X., Hiew S., Brady H., Liu Y., Dou Y. (2009). Dicer independent small RNAs associate with telomeric heterochromatin. *RNA*.

[117] Idda M. L., Munk R., Abdelmohsen K., Gorospe M. (2018). Noncoding RNAs in Alzheimer's disease. *Wiley Interdisciplinary Reviews: RNA*.

[118] Matera A. G., Terns R. M., Terns M. P. (2007). Non-coding RNAs: lessons from the small nuclear and small nucleolar RNAs. *Nature Reviews Molecular Cell Biology*.

[119] Sinha H., Nicholson B. P., Steinmetz L. M., McCusker J. H. (2006). Complex genetic interactions in a quantitative trait locus. *PLoS Genetics*.

[120] Seto A. G., Kingston R. E., Lau N. C. (2007). The coming of age for Piwi proteins. *Molecular Cell*.

[121] Esteller M. (2011). Non-coding RNAs in human disease. *Nature Reviews Genetics*.

[122] Hombach S., Kretz M. (2016). Non-coding RNAs: classification, biology and functioning. *Non-Coding RNAs in Colorectal Cancer, Ed*.

[123] Rasmussen T. P. (2019). Parallels between artificial reprogramming and the biogenesis of cancer stem cells: involvement of lncRNAs. *Seminars in Cancer Biology, Ed*.

[124] Lei B., Tian Z., Fan W., Ni B. (2019). Circular RNA: a novel biomarker and therapeutic target for human cancers. *International Journal of Medical Sciences*.

[125] Ogawa Y., Sun B. K., Lee J. T. (2008). Intersection of the RNA interference and X-inactivation pathways. *Science*.

[126] Yang Z., Jiang X., Jiang X., Zhao H. (2018). X-inactive-specific transcript: a long noncoding RNA with complex roles in human cancers. *Gene*.

[127] Belostotsky D. (2009). Exosome complex and pervasive transcription in eukaryotic genomes. *Current Opinion in Cell Biology*.

[128] Taft R. J., Kaplan C. D., Simons C., Mattick J. S. (2009). Evolution, biogenesis and function of promoter-associated RNAs. *Cell Cycle*.

[129] Taft R. J., Glazov E. A., Lassmann T., Hayashizaki Y., Carninci P., Mattick J. S. (2009). Small RNAs derived from snoRNAs. *RNA*.

[130] Ender C., Krek A., Friedländer M. R. (2008). A human snoRNA with microRNA-like functions. *Molecular Cell*.

[131] Shi W., Hendrix D., Levine M., Haley B. (2009). A distinct class of small RNAs arises from pre-miRNA-proximal regions in a simple chordate. *Nature Structural & Molecular Biology*.

[132] Langenberger D., Bermudez-Santana C., Hertel J., Hoffmann S., Khaitovich P., Stadler P. F. (2009). Evidence for human microRNA-offset RNAs in small RNA sequencing data. *Bioinformatics*.

[133] Thompson D. M., Parker R. (2009). Stressing out over tRNA cleavage. *Cell*.

[134] Xu M., Medvedev S., Yang J., Hecht N. B. (2009). MIWI-independent small RNAs (MSY-RNAs) bind to the RNA-binding protein, MSY2, in male germ cells. *Proceedings of the National Academy of Sciences*.

[135] Carone D. M., Longo M. S., Ferreri G. C. (2009). A new class of retroviral and satellite encoded small RNAs emanates from mammalian centromeres. *Chromosoma*.

